# Recreational freshwater fishing drives non-native aquatic species richness patterns at a continental scale

**DOI:** 10.1111/ddi.12557

**Published:** 2017-06

**Authors:** A. J. S. Davis, J. A. Darling

**Affiliations:** 1 Oak Ridge Institute for Science and Engineering (ORISE), US Environmental Protection Agency, Research Triangle Park, NC, USA; 2 National Exposure Research Laboratory, US Environmental Protection Agency, Research Triangle Park, NC, USA

**Keywords:** aquatic biological invasions, freshwater recreational fishing, macroscale, non-native richness, population density, sampling bias

## Abstract

**Aim:**

Mapping the geographic distribution of non-native aquatic species is a critically important precursor to understanding the anthropogenic and environmental factors that drive freshwater biological invasions. Such efforts are often limited to local scales and/or to single species, due to the challenges of data acquisition at larger scales. Here, we map the distribution of non-native freshwater species richness across the continental United States and investigate the role of human activity in driving macro-scale patterns of aquatic invasion.

**Location:**

The continental United States.

**Methods:**

We assembled maps of non-native aquatic species richness by compiling occurrence data on exotic animal and plant species from publicly accessible databases. Using a dasymetric model of human population density and a spatially explicit model of recreational freshwater fishing demand, we analysed the effect of these metrics of human influence on the degree of invasion at the watershed scale, while controlling for spatial and sampling bias. We also assessed the effects that a temporal mismatch between occurrence data (collected since 1815) and cross-sectional predictors (developed using 2010 data) may have on model fit.

**Results:**

Non-native aquatic species richness exhibits a highly patchy distribution, with hotspots in the Northeast, Great Lakes, Florida, and human population centres on the Pacific coast. These richness patterns are correlated with population density, but are much more strongly predicted by patterns of recreational fishing demand. These relationships are strengthened by temporal matching of datasets and are robust to corrections for sampling effort.

**Main conclusions:**

Distributions of aquatic non-native species across the continental US are better predicted by freshwater recreational fishing than by human population density. This suggests that observed patterns are driven by a mechanistic link between recreational activity and aquatic non-native species richness and are not merely the outcome of sampling bias associated with human population density.

## 1 | INTRODUCTION

Non-native aquatic species (NAS) are significant drivers of ecological change in freshwater ecosystems throughout the world (Strayer, 2010; Ricciardi & MacIsaac, 2011). In the United States, as in many other regions, freshwater biological invasions have been implicated in the precipitous decline of endemic biodiversity (Ricciardi, Neves, & Rasmussen, 1998) and have been shown to cause dramatic losses in a variety of ecosystem services ranging from recreational fishing to water quality (Rothlisberger, Finnoff, Cooke, & Lodge, 2012; Walsh, Carpenter, & Vander Zanden, 2016). Understanding the factors that contribute to current distributions of aquatic invasive species remains an important step towards designing effective public policy aimed at predicting and preventing future invasions and mitigating their ecological and socio-economic costs (Simberloff, Parker, & Windle, 2005).

Anthropogenic activity is known to be a primary driver of biological invasion patterns, with multiple vectors of species introduction operative at different spatial scales. Estimates of propagule pressure (the number of viable individuals introduced to a recipient area over time) that reflect anthropogenic activity, such as population density and distance to major transportation hubs, have been shown to have strong effects on distributions of non-native species (Gallardo, Zieritz, & Aldridge, 2015; Bellard, Leroy, Thuiller, Rysman, & Courchamp, 2016). Furthermore, trends in global trade have shaped geographic and taxonomic patterns of biological invasions for centuries (Westphal, Browne, MacKinnon, & Noble, 2007; Hulme, 2009), and intercontinental translocation of NAS continues to accompany vectors such as international vessel traffic, aquaculture and aquarium trade (Padilla & Williams, 2004; Lo, Levings, & Chan, 2012; Cope, Prowse, Ross, Wittmann, & Cassey, 2015).

In addition, the secondary spread of established NAS has been strongly linked to recreational vectors such as boating and sport fishing, through mechanisms including hull and equipment fouling and bait transfer (Rothlisberger, Chadderton, McNulty, & Lodge, 2010; Clarke Murray, Pakhomov, & Therriault, 2011; Nathan, Jerde, Budny, & Mahon, 2014). Unfortunately, because they generally rely on costly surveys of boaters for data collection, efforts to understand the role of recreational activity in driving freshwater invasions have been conducted on limited scales, primarily targeting risk assessment for particular invasive species (Buchan & Padilla, 2000; Bossenbroek, Kraft, & Nekole, 2001; Wittmann, Kendall, Jerde, & Anderson, 2015) or focusing on development of regional models (Muirhead & Macisaac, 2005; Leung, Bossenbroek, & Lodge, 2006; Chivers, Leung, & Matthiopoulos, 2012). Similar limitations exist for continental-scale NAS distribution data. The expense associated with extensive sampling for NAS typically precludes coordinated monitoring and data collection efforts even at the regional level, thus forestalling opportunities to investigate macroscale patterns and drivers of non-native species richness. These limitations have thus far largely precluded investigation of potential broader effects of recreational activity on exotic aquatic species richness patterns, despite the fact that extensive continental-scale transportation networks provide plausible mechanisms linking recreational boating and fishing to invasive spread at the macroscale (Bossenbroek, Johnson, Peters, & Lodge, 2007).

Ad hoc repositories of biodiversity data resulting from the compilation of multiple sources of occurrence records including herbarium specimens and scientific and opportunistic surveys conducted by federal and state agencies, universities, non-governmental organizations and citizen scientists are a promising alternative to the large-scale collection of primary occurrence data. Despite the availability of such databases, to the best of our knowledge, there is no single comprehensive spatial database of freshwater non-native species occurrence data for the continental United States (CONUS), and no tool presently exists that readily allows the visualization of macroscale invasion patterns based on overall species richness. This limitation prevents comprehensive continental-scale mapping of freshwater invasion hotspots. Studies conducted for terrestrial species (Liebhold et al., 2013; Iannone et al., 2015) and for fish (Stohlgren et al., 2006) have demonstrated the utility of such broad-scale analyses of exotic species richness for understanding both anthropogenic and environmental drivers of biological invasions in the United States. However, potential sources of spatial, temporal and sampling bias arising from the use of ad hoc databases are frequently not addressed. Failure to address these potential biases can lead to erroneous inferences, in extreme cases even generating results reversing the true direction of ecogeographical relationships (Ficetola et al., 2014).

In this study, we summarize geographic patterns of NAS richness across the CONUS, utilizing data drawn from multiple publicly available sources. Compilation of occurrence data for a large number of non-native plant and animal species allows us to map hotspots of NAS richness and to examine macroscale distribution patterns across taxa. Using these data, we tested the hypothesis that human activity has driven continental-scale patterns of freshwater invasion. To do this, we take advantage of two unique datasets related to the human footprint. The first is a dasymetric map of US population density, which is a realistic reallocation of human population estimates from the US 2010 census block group level to the 30 m pixel level based on land use characteristics. This dataset removes the boundary effect imposed by census block groups and allows population density to be calculated using appropriate ecological units, that is, watersheds. The second is a national model of the distribution of freshwater recreational fishing demand (Mazzotta, Wainger, Sifleet, Petty, & Rashleigh, 2015). This model allows, for the first time, continental-scale assessment of the role that recreational fishing plays in shaping patterns of freshwater biological invasions. Given widely recognized correlations between metrics of human influence and non-native species richness (Gallardo et al., 2015), we hypothesized that both population density and recreational demand would predict overall NAS richness. However, based on the assumption that freshwater fishing demand is a more direct proxy of human-mediated propagule pressure than population density, we expected freshwater fishing demand to be the superior predictor. We also examined the effect of correcting for potential sources of spatial and temporal bias, respectively, using spatial eigenvectors and using temporally matched data obtained by subsetting NAS occurrence data so that it more closely approximates the timeframe of the predictors. Finally, to ensure that our findings were not solely the result of sampling bias, we used species rarefaction to correct for varying sampling effort and assessed whether our findings were robust to these corrections.

## 2 | METHODS

### 2.1 | Database development

We restricted our database to those species that are non-native to the United States. Species introduced outside their primary US geographic range to other regions in the United States (commonly referred to as “native transplants”) were excluded. We applied this definition to the non-indigenous animal species listed by the US Geological Survey’s Non-indigenous Aquatic Species program (USGS-NAS) that inhabit freshwater habitats to obtain a list of 287 species. We compiled a non-native freshwater aquatic plant species list (*n* = 65) from the Invasive Plant Atlas of the United States (maintained by the University of Georgia’s Center for Invasive Species and Ecosystem Health) and the US Army Corps of Engineers’ Aquatic Plant Management Information System. Using these lists, we compiled non-native species occurrence data from USGS-NAS, the Early Detection and Distribution Mapping System (EDDMapS) and the USGS Biodiversity Serving Our Nation (BISON) database, which is the US data repository for the Global Biodiversity Information Facility (GBIF). As at the time of this analysis, the USGS-NAS database was limited to animal species, our plant data were extracted only from the latter two sources. Duplicate occurrence records, centroids, records with missing spatial coordinates and occurrence data outside the CONUS were removed. The remaining occurrence records were georeferenced to USGS hydrological unit code 8 (HUC 8) using ARCGIS (v. 10.3.1). The hydrological unit code system is a hierarchical cataloguing system of nested watersheds delineated using topographic and hydrological features; HUC 8s represent surface drainage basins. We selected HUC 8s for this analysis to facilitate compatibility with the USGS-NAS program. There were a total of 2106 HUC 8 watersheds in our study extent, with an average size of 1,821 km^2^. To assess the effects of potential temporal bias arising from substantial temporal mismatch with the cross-sectional predictors measured in 2010 (described below) and the occurrence data, which contain species that have been reported in some locations since the early 19th century, we also extracted a temporal subset of plant and animal richness data using only occurrence data that were reported beginning in 2005 and ending when the data were extracted (October–November 2015) from the database. This temporal subset is referred to as the 2005+ subset. Our final database of non-native aquatic biodiversity represents a total of 264 species (202 animals and 62 plants), with 181,110 georeferenced observations recorded between 1815 and 2015. The 2005+ subset contained a total of 112,712 observations spanning 191 species (137 animals and 54 plants; see Supporting Information, Appendix S1).

### 2.2 | Distribution maps

We created plant and animal richness distribution maps summarized at the HUC 8 level across the continental United States to visualize broad-scale invasion patterns. Richness was calculated simply as the total number of unique non-native species observed at least once in the hydrological unit. The Getis–Ord General G was employed using the Spatial Statistics toolbox in ARCGIS (v.10.3.1) to reveal the presence of statistically significant clusters of high or low non-native species richness.

### 2.3 | Dasymetric population mapping and freshwater fishing demand

The 2010 census block data were allocated to 30 m^2^ pixels on the basis of the 2011 National Land Cover Dataset (NLCD; Homer et al., 2015) and slope to create a dasymetric population map reflecting the most probable distribution of humans for the CONUS. Likely uninhabitable areas such as open water or slopes exceeding 25% were excluded during the allocation process (Mennis & Hultgren, 2006; Mazzotta et al., 2015). Using this layer, we obtained the number of people estimated to reside in each HUC 8 watershed and divided this by the area of the HUC 8 to calculate population density in km^2^.

We utilized a model of freshwater recreational demand previously described by Mazzotta et al. (2015). Briefly, the US Fish and Wildlife Service’s National Survey of Fishing, Hunting, and Wildlife-Associated Recreation (FHWAR) data were linked to the dasymetric population data and estimates of willingness to travel based on the US Forest Service National Visitor Use Monitoring (NVUM) programme. A probability density function was used to estimate the number of days that freshwater fishing was demanded in each 30 m^2^ pixel, based on population distribution, demographic specific participation rates and travel distance. Using this model, we derived the cumulative sum of fishing demand for each HUC 8 and normalized by area in km^2^. The model does not necessarily reflect recorded recreation, but rather predicts expected demand for freshwater fishing based on available data. For further details on development of the demand model, see Mazzotta et al. (2015). Both datasets are available to the public through the US Environmental Protection Agency’s EnviroAtlas at https://www.epa.gov/enviroatlas/enviroatlas-data (Pickard, Daniel, Mehaffey, Jackson, & Neale, 2015).

### 2.4 | Data analyses

**FIGURE 1:**
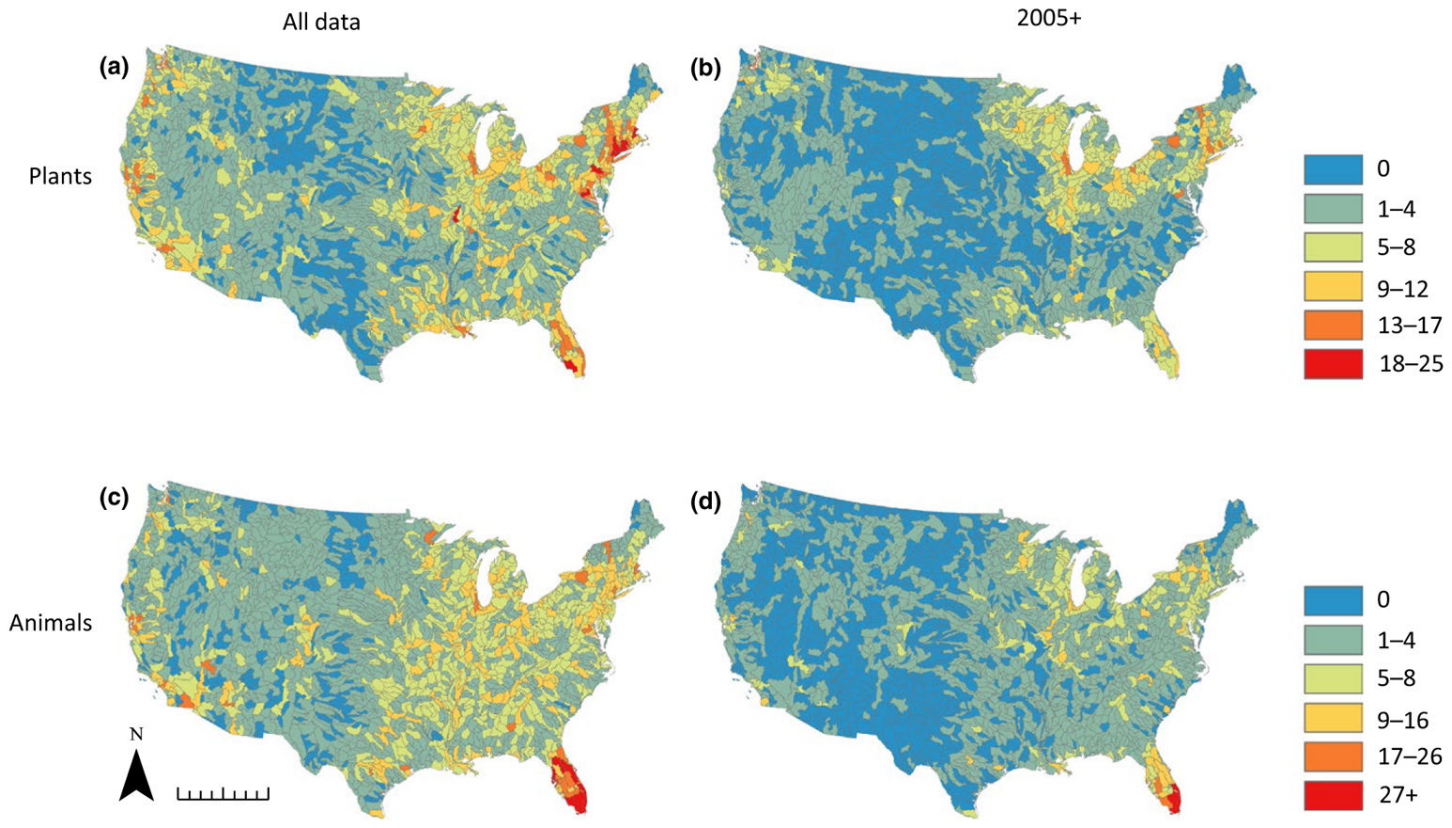
Distribution of non-native aquatic species richness across the continental United States. Maps showing non-native plant richness (a, b) and animal richness (c, d), summarized at HUC 8 hydrological units for both the complete dataset (a, c) and the subset of data from 2005 to present (b, d). Note that the colour scale for plants differs from those for animals. Scale bar at bottom left indicates 1,000 km. [Colour figure can be viewed at wileyonlinelibrary.com]

**FIGURE 2:**
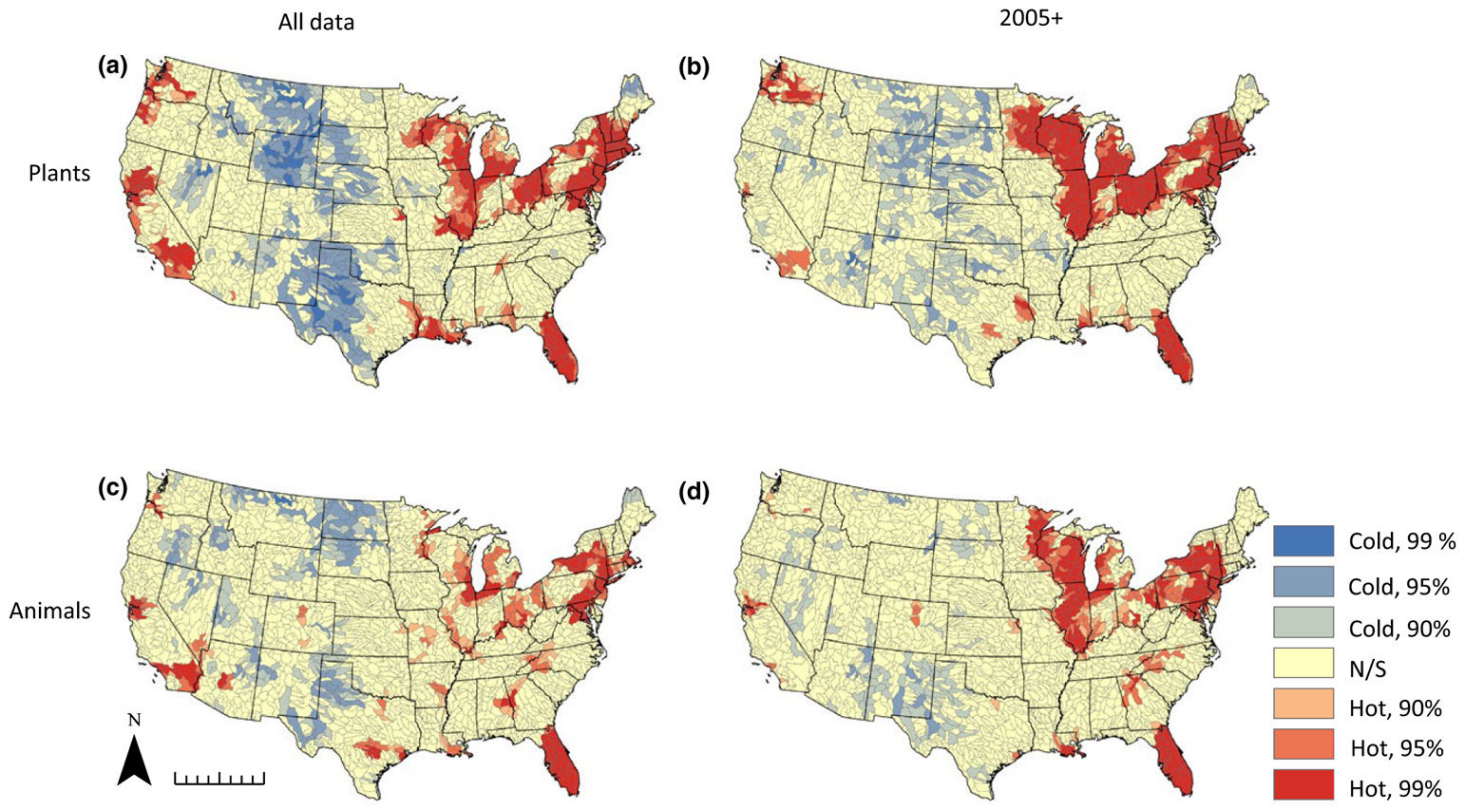
Map of HUC 8 clusters with highest (red) and lowest (blue) concentrations of non-native plant richness (a,b) and animal richness (c,d) for both the complete dataset (a,c) and the subset of data from 2005 to present (b,d). Colours indicate confidence at the 90%, 95% and 99% levels as indicated in the legend. N/S, non-significant; scale bar at bottom left indicates 1,000 km. [Colour figure can be viewed at wileyonlinelibrary.com]

We compared the effect of freshwater fishing demand and human population density on animal and plant non-native species richness, respectively, using generalized linear models (GLM) with a quasi-Poisson distribution to account for overdispersion (Crawley, 2007). To account for the varying amount of aquatic habitat available in each watershed, the percentage of each HUC 8 covered by open water or wetlands as indicated in the 2011 NLCD was used as an offset in the models. Predictors were mean-centred and standardized. As is evident in Figures 1 and 2, highly invaded watersheds tended to cluster together, suggesting that the data are spatially structured. This is not unexpected, as invasive species patterns aggregate as a result of local dispersal, and also in response to spatial structuring that is often present in the explanatory variables (Legendre, 1993; Bahn, Krohn, & O’Connor, 2008). We used Moran’s *I* to confirm the presence of spatial autocorrelation in the residuals of preliminary models (Table 1). Spatial Poisson models were constructed using spatial eigenvectors derived from the data to remove the effects of spatial dependence from regression residuals as described by Griffith and Peres-Neto (2006). We reported exponentiated coefficients and their 95% confidence intervals (CI) to provide an interpretation of effect size in the form of incidence rate ratios (IRR). IRRs indicate the expected rate of increase in the response variable for every one unit increase in the predictor of interest, holding all others constant. Quasi-AIC (QAIC) uses a scale parameter, c-hat, to rescale the model likelihoods used to estimate Akaike’s information criterion (AIC) in order to accommodate overdispersed data (Anderson, Burnham, & White, 1994). We used a nested approach to compare QAIC of spatial and aspatial models, where the spatial model was treated as the global model (the model with the full set of predictors) from which we derived c-hat, and the aspatial model is a subset of the global model.

Species richness estimates are often strongly correlated with sampling effort (Gotelli & Colwell, 2001). In our data, species richness and sampling effort (measured as the total number of records for each watershed) were significantly positively correlated for both plants (all data: *r* = .72; 2005+: *r* = .63, *p* < .05) and animals (all data: *r* = .58; 2005+: *r* = .60, *p* < .05). To ensure that our findings were not biased by uneven sampling effort, we used species rarefaction implemented with the INEXT R package (Hsieh, Ma, & Chao, 2016) to estimate the species richness that would be present in watersheds if all watersheds had received the same level of sampling effort. Prior to rarefaction, watersheds with at least 10 occurrences were selected from the full dataset and the 2005+ temporal subset. This decreased the sample size from 2,106 watersheds to 1,253 watersheds with the minimum sample size for animals and 833 watersheds for plants using the full dataset, and 536 and 518 watersheds for animals and plants, respectively, using the 2005+ subset. Simultaneous autoregressive (SAR) models were used to analyse the effects of freshwater fishing demand and population density on rarified animal and plant richness. SAR models were adopted instead of spatial Poisson models to account for spatial autocorrelation in the data as the species rarefaction transforms richness data from count to continuous.

## 3 | RESULTS

The distribution maps of non-native species richness based on the full dataset reveal that the eastern half of the United States and the western coastal states (Washington, Oregon and California) harbour the highest levels of reported NAS for both plant and animal species (Figure 1). The Great Plains and Mountain West exhibit markedly lower non-native richness, with the majority of watersheds in these regions harbouring one to four species. Distribution maps created using the 2005+ subset show a reduction in overall reported non-native species, consistent with inclusion of only a fraction of the total sampling effort represented by the entire dataset. Plant and animal species invasion patterns remain aggregated in the Great Lakes region, New England and Florida, with lower levels of richness in the Pacific coastal states. Hotspot analyses reveal statistically significant clusters of high non-native species richness in the Great Lakes region, New England and Florida in both the full datasets and the 2005+ subsets (Figure 2). Invasion cold spots are generally restricted to the Great Plains, Mountain West and the region encompassing west Texas, Oklahoma and New Mexico in both the overall and 2005+ datasets. Despite differences between the full dataset and the 2005+ subset, general continental-scale patterns remain largely consistent. Also, plant animal and animal richness exhibit similar geographical clustering as indicated by the hotspot analyses, with the exception of the presence of animal richness hotspots in the southern Appalachian region in western North Carolina, South Carolina and Georgia; this pattern is more pronounced in the 2005+ dataset (Figure 2).

**FIGURE 3:**
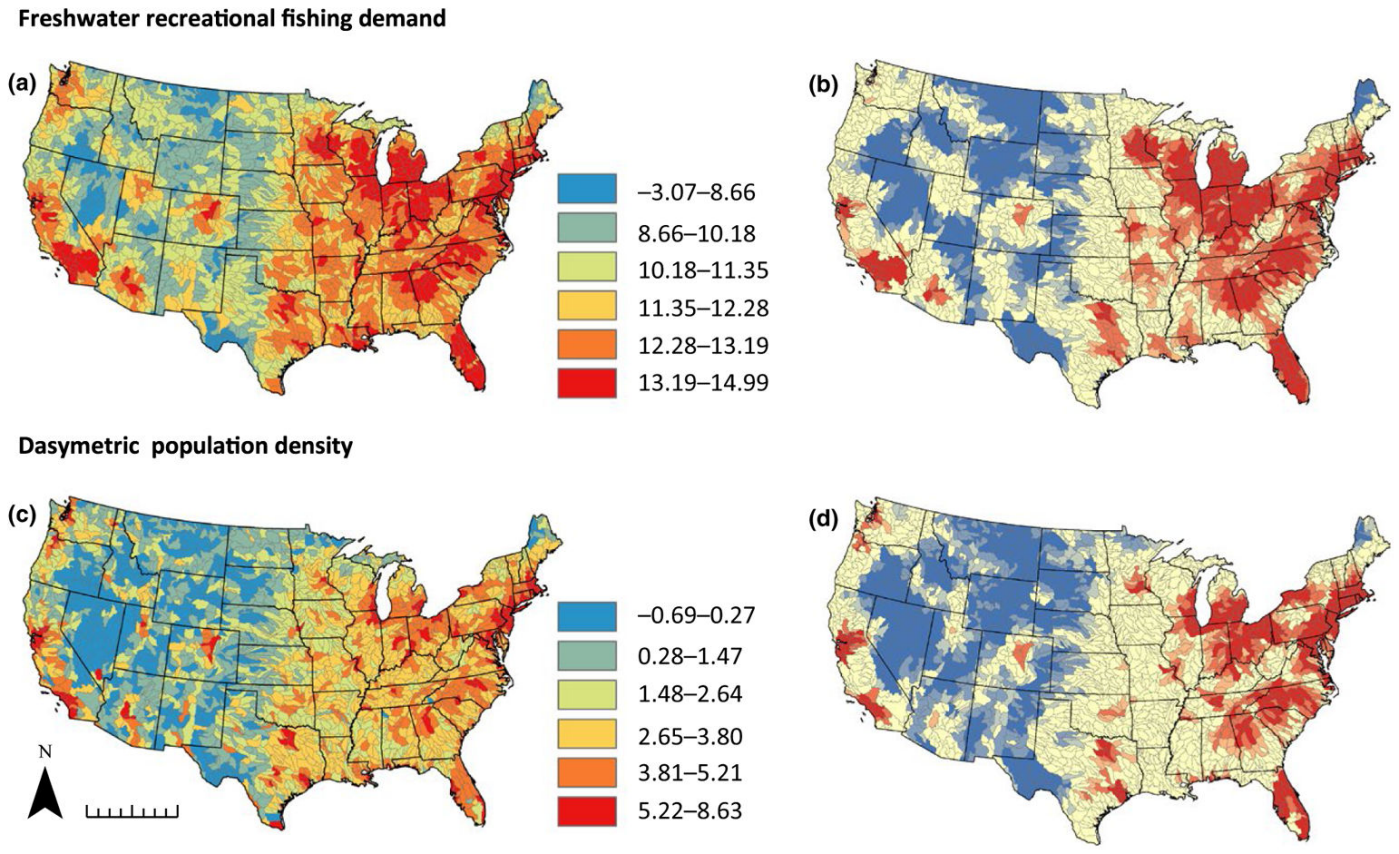
Raw data (a, c) and hotspot analysis (b, d) for freshwater recreational fishing demand (a, b) and dasymetric population density (c, d). Both predictors have been mean-centred and standardized. Colour scale shown is for raw data only; colour scale for hotspot analysis is as in Figure 2. All data are summarized at the eight-digit HUC. Scale bar at bottom left indicates 1,000 km. [Colour figure can be viewed at wileyonlinelibrary.com]

General patterns of NAS richness visually align closely with patterns of both freshwater recreational fishing demand and dasymetric population density (Figure 3). Both metrics show aggregations in the eastern United States and along the Pacific coast, and hotspot analysis shows similar patterns of high population density and recreational demand in the Great Lakes, along the Atlantic seaboard and near population centres in California and the Pacific Northwest. Unlike non-native species richness, both population and recreational demand show significantly elevated values in the south-eastern United States outside Florida.

The results of the quasi-Poisson models confirm that both population density and freshwater fishing demand are statistically significant predictors of non-native aquatic richness (Table 1). Although this holds true for both aspatial and spatial models, the spatial eigenvector method was effective in reducing Moran’s *I* to inconsequential levels in the spatial models and accounting for spatial autocorrelation resulted in a much better model fit as indicated by QAIC (Table 2). Our results indicate that models with human population density as the predictor had higher spatial autocorrelation, as indicated by Moran’s *I*, than models with freshwater fishing demand (Table 1). After controlling for spatial autocorrelation, the model coefficients for human population density on non-native animal and plant richness decreased, whereas freshwater fishing demand resulted in a minimal decrease in the coefficients for animal and plant richness. This indicates that the relationship between human population density and NAS richness is more influenced by the spatial structure (spatial sampling bias) of the predictor, while the relationship between non-native species richness and freshwater fishing demand is more robust.

Overall, model fits were also improved when analyses were conducted with the 2005+ temporal subset of data (Table 2). In all models, freshwater fishing demand proved to be a stronger predictor of both animal and plant richness than did population density (Table 1). Although this difference was not significant in aspatial models utilizing the full dataset (as indicated by overlapping CIs on IRR), improvements in model fit through correction for either spatial autocorrelation or temporal matching of the data resulted in significantly higher effect sizes for models using freshwater fishing as the predictor. In the strongest models (spatial models utilizing the 2005+ dataset), effect sizes for freshwater fishing were 20.4% higher than population density for non-native animal species and 25.9% higher for plant species, with non-overlapping CIs.

Species rarefaction substantially reduced the correlation between animal richness and sampling efforts (all data: *r* = −.09, *p* > .05; 2005+: *r* = −.03, *p* > .05), although it did introduce significant negative correlation with plant richness in the 2005+ subset (all data: *r* = .05, *p* > .05; 2005+ : *r* = −.31, *p* < .05). The results of SAR models generally support the findings of quasi-Poisson models of the non-rarified dataset. Freshwater fishing demand remains a better predictor than population density of rarified animal and plant richness obtained from the 2005+ subset, having both a higher Nagelkerke *R*^2^ and lower AIC (Table 3). Two-sample *z* tests of the means indicated that the differences in coefficients for freshwater fishing and population density are statistically significant (*z* = 5.1, *p* = <.001 for animals; *z* = 6.2, *p* = <.001 for plants). The results from the full dataset indicated that while rarified plant richness was better predicted by freshwater fishing demand, again with a statistically significant difference in coefficients (*z* = 5.8 *p* = <.001), that relationship did not persist for rarified animal richness. In all models, both population density and freshwater fishing remained significant predictors (*p* < .001) of NAS richness.

## 4 | DISCUSSION

Distribution patterns of NAS clearly indicate an important role for human activity in driving NAS introductions and translocations at a continental scale, consistent with past observations of human influence as a macroscale driver of biological invasions. Hotspots of NAS richness (Figures 1 and 2) reflect similar patterns observed elsewhere for several non-native taxonomic groups. For instance, previous examination of forest pest invasions across the CONUS found them to be predominantly aggregated in the north-eastern United States, suggesting that combined effects of propagule pressure (reflected in metrics of human influence such as population density) and habitat invasibility shaped the current distribution (Liebhold et al., 2013). Similarly, Stohlgren et al. (2006) observed clusters of non-native plant diversity in the Northeast, Great Lakes, Florida and Pacific coast states. Interestingly, significant departure of our findings from previous descriptions of non-native fish distributions is largely due to differences in the Mountain West (Stohlgren et al., 2006), where considerably higher fish richness in earlier studies likely reflects the inclusion of native transplants and the longstanding practice of fish stocking in that region (Pister, 2001). The observed macroscale patterns generally comport with expectations based on historical trends in vector activity. The north-eastern United States not only exhibits high current population density but historically has been a population centre for longer than any other region, with a correspondingly longer history of activities associated with high propagule pressure (Reichard & White, 2001). The Great Lakes are widely recognized as a global hotspot of freshwater biological invasions, with accompanying invasion pressure extending to the surrounding region (Muirhead & Macisaac, 2005; Ricciardi, 2006). Also worth noting in the current study is the degree of NAS richness observed in Florida, which for animals in particular was far higher than any other region. This finding is unsurprising, given that Florida represents the largest centre of exotic pet trade in the United States (Krysko et al., 2011; Fukisaki, Mazzotti, Watling, Krysko, & Escribano, 2015). These observations draw attention to the potential importance of vectors other than recreational activities driving NAS distributions, and especially to the possibility that vectors associated with initial introduction may be distinct from those driving secondary spread. In the Great Lakes region, for instance, recreation many be an important secondary driver for many species initially introduced to the United States through international ballast water transport (Ricciardi, 2006). Similarly, the pet and aquarium trades have resulted in introductions of a variety of NAS subsequently spread widely by other mechanisms (Padilla & Williams, 2004; Krysko et al., 2011). Our analysis thus may not capture the role that these initial introduction vectors may play in shaping the existing distribution of NAS in the United States.

The NAS distribution maps underscore the value of recognizing NAS hotspots to inform prioritization of conservation effort and support design of effective NAS mitigation policy. Approaches to identifying native biodiversity hotspots have formed the basis for conservation efforts at global and continental scales, grounded in the understanding that disproportionately high diversity offers opportunities to leverage limited resources to greatest effect (Myers, Mittermeier, Mittermeier, Fonseca, & Kent, 2001). Similarly, hotspot analysis has been utilized to evaluate the likely effectiveness of management approaches aimed at curtailing risks associated with aquatic invasions (Drake & Lodge, 2004), and to determine potential threats posed by non-native species to ecologically sensitive systems and native biodiversity (Adhikari, Tiwary, & Barik, 2015). The results presented here thus represent a resource for future appraisal of policy and management options. For instance, in addition to enabling identification of heavily invaded regions, our analysis also identifies regions that may be unusually underinvaded relative to expectations. Specifically, areas in the south-eastern United States outside Florida (e.g., parts of VA, NC, SC, TN and GA) are high-growth areas that represent both hotspots of population density and recreational fishing demand, but are not currently associated with high levels of non-native richness in our dataset (Figures 2 and 3). This suggests either that watersheds in these areas are already highly invaded but are undersampled, or that they may be at increased risk of future invasion due to heightened propagule pressure associated with anthropogenic drivers. Either prospect is troubling given that this region also represents a centre of native aquatic diversity, one that may be insufficiently protected by current conservation efforts (Jenkins, Van Houtan, Pimm, & Sexton, 2015). The presence of a small hotspot of non-native animal diversity in the southern Appalachian region (western NC, SC and north Georgia, Figure 2) suggests that this region of endemic diversity may already be experiencing a growing threat from aquatic invasions.

Statistical analyses strongly support our hypothesis that recreational freshwater fishing demand is an important driver of the observed NAS distribution across the CONUS, thus bearing out anticipated relationships between measures of human activity and NAS richness. Propagule pressure is generally appreciated as perhaps the most critical consideration in explaining invasion patterns and estimating invasion risk (Lockwood, Cassey, & Blackburn, 2005; Von Holle & Simberloff, 2005). Unfortunately, it is also difficult or impossible to measure directly for most systems. As a result, studies typically employ more readily obtainable proxy measures of propagule pressure. The utility of these metrics depends on a close relationship between the proxy and actual propagule pressure, which may be more tenuous for some proxies than it is for others (Wonham, Byers, Grosholz, & Leung, 2013). Proxies adopted to understand risks associated with NAS have ranged broadly, depending on the scale of analysis. While national- and global-scale data are often available for commercial vectors (e.g., ballast water volume (Chan, Bailey, Wiley, & MacIsaac, 2012), commercial ship visits (Drake & Lodge, 2004; Keller, Drake, Drew, & Lodge, 2011; Lo et al., 2012), ship tonnage (Cope et al., 2015)), variables used to explore the relationships between recreational fishing and boating and NAS risk have generally been limited in geographic scope due to the cost and effort associated with collecting data (Buchan & Padilla, 2000; Muirhead & Macisaac, 2005; Leung et al., 2006; Clarke Murray et al., 2011; Wittmann et al., 2015). Macroscale analyses of these relationships are nevertheless crucial, given the fact that recreational vector connectivity at continental scales poses substantial and sometimes predictable risks of invasive spread (Bossenbroek et al., 2007). The freshwater recreational demand model employed in our study represents a novel opportunity to explore such macroscale relationships. In our analyses, the modelled pattern of freshwater fishing demand strongly predicts distribution of NAS species richness across the CONUS. The strength of this predictor is significantly greater than that of population density alone (Table 1), providing further evidence that proxies of propagule pressure that more closely reflect the mechanistic relationship between human activity and NAS spread are likely to improve the accuracy of species distribution models and risk assessments (Bradie, Chivers, Leung, & Richardson, 2013; Davis, Singh, Thill, & Meentemeyer, 2016). To the best of our knowledge, this study is the first to provide empirical evidence linking recreational fishing activity with reported NAS richness patterns at the continental scale.

Although freshwater fishing demand is a statistically better predictor than population density, the latter clearly remains a useful, if less direct, proxy of propagule pressure in aquatic systems. Fishing demand and dasymetric population density have a strongly linear relationship (*r* = .76, *p* < .05 when both predictors are log-transformed, see Supporting Information, Figure S1), as expected given that the latter is an input to the freshwater recreational demand model. Notable outliers in this correlation generally represent areas with high fishing demand but low population density, suggesting that while water resources near population centres universally receive high recreational pressure, the opposite may not hold true; there are a number of “destination” watersheds far from dense populations that nevertheless are expected to experience high recreational demand. These patterns highlight the fact that, despite their strong correlation, recreational demand and population density likely represent different clusters of anthropogenic dispersal vectors that may have differential effects on dispersal at the species level. The causal link between recreational fishing and dispersal of any particular NAS obviously depends on the characteristics of that species, although it is worth noting that recreational fishing represents a broad suite of vectors widely recognized to be generally important in the spread of aquatic invasives, including fouling of trailered boats and other equipment (Rothlisberger et al., 2010) as well as bait bucket transfers (Nathan et al., 2014). While the specific aim in the current study was to test explicit hypotheses regarding the strength of different proxy variables in predicting macroscale distribution patterns, future research could build stronger predictive models by incorporating additional data on other likely drivers of NAS establishment and spread. In addition to a wide variety of possible natural and human dispersal vectors, myriad other anthropogenic and environmental factors also likely influence NAS distributions, with the strength of effect strongly dependent on biological and ecological characteristics of individual species. Establishment and spread of NAS can be expected to respond to factors as varied as climate (Rahel & Olden, 2008), water chemistry (Whittier, Ringold, Herlihy, & Pierson, 2008), hydrological alteration (Catford et al., 2014) and pollution (Varo et al., 2015). Unfortunately, very few of these factors have consistent data coverage across the CONUS.

Our analyses have been made possible only through the rapidly increasing availability of digital biodiversity information. While the accessibility of such information offers unprecedented opportunities for understanding broad-scale patterns of both native and non-native biodiversity, the ad hoc nature of these resources does recommend caution. For example, patterns of species richness such as those observed in the current dataset may be vulnerable to taxonomic errors and geographic location inaccuracies, which are limitations of publicly available species distribution data (Maldonado et al., 2015) that can be largely overcome with careful data checking and cleaning (Boakes et al., 2010) such as employed in preparation of our NAS database. The accumulation of data over large timescales, typical of digital bio-diversity repositories, generates additional challenges for assessing the importance of potential drivers of non-native species distributions (Liebhold et al., 2013). In our analyses, the strength of the recreational demand predictor relative to population density increased when utilizing NAS occurrence data temporally matched to the timeframe of the predictor variables (Table 1). This suggests that temporal mismatch between occurrence data and potential predictors could decrease the power to detect significant relationships, a phenomenon that may warrant further study.

Another difficulty associated with utilizing ad hoc databases of non-native species occurrence is the fact that correlations between human population density and NAS richness can arise from population density-induced sampling bias, in which increasing population density provides greater opportunity to observe invasions (Gallardo et al., 2015; Bellard et al.,2016). Such bias can result in geographic agglomerations of species observations induced by the spatial structure of human population density, in which watersheds with higher population density are those that are also likely to receive higher sampling effort and thus be spatially autocorrelated. In this study, the use of spatial models allowed us to control for the effects of spatial autocorrelation induced by this potential positive sampling bias, so as to not overestimate the effects of predictors on non-native species richness. Accounting for spatial autocorrelation not only improved overall model fit but also further increased the strength of the freshwater fishing demand predictor relative to population density.

Species rarefaction methods (Chao et al., 2014) represent an alternative means of correcting sampling bias. This method can be used to address undersampling bias (Gottelli and Colwell, 2011), in which the overall number of individuals sampled in some regions is relatively small compared to better sampled areas, leading to correspondingly small estimates of species richness. These and similar approaches have been developed for analysis of communities dominated by native species and therefore not expected to respond positively to anthropogenic drivers. However, in the case of non-native species, population density or other metrics of human activity are expected to correlate not only with sampling bias but also with the distribution of non-native species *via* propagule pressure. Therefore, attempts to remove the observer effect arising from population density-induced sampling bias may substantially impair ability to detect relationships between human activity and NAS richness. Indeed, the effects of species rarefaction on inferences with non-native occurrence data gathered from ad hoc databases are largely unknown. Also problematic for exploring non-native species richness is the culling of data that are inherent to rarefaction approaches. In this study, low numbers of NAS records in many watersheds resulted in reductions in sample size of between 40% and 75% after rarefaction, depending on the model. Species rarefaction therefore represents an aggressive approach to correcting for sampling effort in studies of non-native diversity. Nevertheless, population density and freshwater fishing demand were observed to be significant predictors of both non-rarified and rarified plant and animal NAS richness in our study, offering strong confirmation that anthropogenic activity represents an important driver of NAS distribution at a continental scale. Also, with the exception of animal richness based on the full dataset, all response variables were again better predicted by freshwater fishing demand than by population density, even after rarefaction. Although these differences were more modest than those observed with non-rarefied richness data, their persistence indicates the robustness of our results to aggressive corrections for sampling bias. Overall, our results demonstrate the possibility of accounting for spatial sampling bias associated with population density without removing important effects of anthropogenic drivers on NAS distribution.

## 5 | CONCLUSIONS

Publicly available species occurrence data are providing novel opportunities to explore the relationships between non-native biodiversity, drivers of invasive spread and resources potentially impacted by biological invasions. Our analyses demonstrate the utility of such data for testing hypotheses regarding the importance of anthropogenic factors in shaping current distributions of non-native richness in freshwater aquatic systems. Specifically, our results support the hypothesis that freshwater recreational fishing demand shapes NAS distributions across the continental United States and that this proxy measure of propagule pressure is a better predictor of those distributions than population density. Our results thus confirm and extend previous single-taxon studies recognizing the importance of anthropogenic propagule pressure in driving patterns of invasion and underscore the necessity of identifying proxy measures that exhibit more direct mechanistic linkages with actual invasive spread. The maps of NAS richness presented here, along with the results of statistical analyses, represent a resource for future investigations of other potential climatic, landscape and environmental drivers of NAS distribution and spread.

## Supplementary Material

AppS1

Sup Fig1

## Figures and Tables

**TABLE 1:** Results from aspatial and spatial quasi-Poisson models comparing freshwater fishing demand with population density on plant and animal richness obtained using the 2005+ temporal subset, and all data

	Aspatial models			Spatial models		
	
	IRR (95% CI)	*df*	Moran’s *I*	IRR (95% CI)	*df*	Moran’s *I*
All data						

Animals						

Population density	1.77 (1.71, 1.82)	2,104	0.19	1.68 (1.64, 1.74)	2,071	0.05

Freshwater fishing	1.81 (1.74, 1.88)	2,104	0.14	1.80 (1.73, 1.87)	2,083	0.05

Plants						

Population density	1.73 (1.67, 1.79)	2,104	0.28	1.57 (1.52, 1.62)	2,065	0.03

Freshwater fishing	1.80 (1.73, 1.87)	2,104	0.24	1.76 (1.69, 1.85)	2,070	0.04

2005+						

Animals						

Population density	2.04 (1.94, 2.13)	2,104	0.26	1.86 (1.77, 1.95)	2,073	0.05

Freshwater fishing	2.38 (2.24, 2.52)	2,104	0.18	2.24 (2.10, 2.39)	2,085	0.06

Plants						

Population density	1.92 (1.83, 2.02)	2,104	0.37	1.62 (1.55, 1.70)	2,065	0.04

Freshwater fishing	2.25 (2.12, 2.39)	2,104	0.30	2.04 (1.91, 2.18)	2,070	0.05

IRR, incidence rate ratio; CI, confidence interval; *df*, degrees of freedom.

All coefficients were significant at *p* < .001.

**TABLE 2:** Nested QAIC results for spatial and aspatial models. The QAIC (quasi-AIC) is Akaike’s information criterion modified to accommodate overdispersed count data. Note that *K* represents the number of parameters for spatial models, all aspatial models are *K* = 2

Model	*K*	c-hat	QAIC	
			Aspatial	Spatial
All data				
Animals				
Population density	35	1.53	6,436.13	5,915.84
Freshwater fishing	23	1.81	5,653.61	5,318.00
Plants				
Population density	41	1.60	6,153.66	5,462.27
Freshwater fishing	36	1.79	5,602.74	5,094.51
2005+				
Animals				
Population density	33	1.27	5,581.96	4,847.04
Freshwater fishing	21	1.43	4,914.44	4,541.21
Plants				
Population density	41	1.41	5,609.49	4,517.54
Freshwater fishing	36	1.53	5,063.06	4,332.71

**TABLE 3:** Results of simultaneous autoregression models of the effect of freshwater fishing demand and dasymetric population density on rarified plant and animal richness

	Std *B*	*SE*	Nagelkerke *R*^2^	AIC	*df*	*z* value	Pr (>|*z*|)
All data							
Rarified animal richness							
Population density	0.10	0.01	0.34	1,238.50	1,248	7.78	<.001
Freshwater fishing	0.13	0.02	0.34	1,248.10	1,248	7.14	<.001
Rarified plant richness							
Population density	0.12	0.02	0.39	883.20	828	6.99	<.001
Freshwater fishing	0.20	0.02	0.41	848.55	828	9.42	<.001
2005+							
Rarified animal richness							
Population density	0.13	0.02	0.32	623.53	531	6.22	<.001
Freshwater fishing	0.18	0.02	0.35	600.28	531	8.01	<.001
Rarified plant richness							
Population density	0.16	0.02	0.29	655.22	513	6.94	<.001
Freshwater fishing	0.25	0.02	0.35	610.42	513	10.00	<.001
