# Supplementary material for: Recreational freshwater fishing drives non-native aquatic species richness patterns at a continental scale
Source: Divers Distrib. Author manuscript; Available in PMC 2018 Aug 22. (PMC6104646; doi:10.1111/ddi.12557)
Supplement: AppS1 [file NIHMS983329-supplement-AppS1.docx]

APPENDIX S1: SPECIES LISTS

| **Plants** |
| --- |
| *Alternanthera philoxeroides* |
| *Alternanthera sessilis* |
| *Arundo donax* |
| *Azolla pinnata* |
| *Butomus umbellatus* |
| *Callitriche stagnalis* |
| *Carex acutiformis* |
| *Ceratopteris thalictroides* |
| *Colocasia esculenta* |
| *Cyperus entrerianus* |
| *Dichanthelium acuminatum* |
| *Egeria densa* |
| *Eichhornia azurea* |
| *Eichhornia crassipes* |
| *Fallopia japonica* |
| *Glyceria maxima* |
| *Heracleum mantegazzianum* |
| *Hydrilla verticillata* |
| *Hydrocharis morsus-ranae* |
| *Hydrocleys nymphoides* |
| *Hygrophila polysperma* |
| *Hymenachne amplexicaulis* |
| *Ipomoea aquatica* |
| *Iris pseudacorus* |
| *Limnophila sessiliflora* |
| *Lysimachia nummularia* |
| *Lysimachia vulgaris* |
| *Lythrum salicaria* |
| *Marsilea mutica* |
| *Marsilea quadrifolia* |
| *Monochoria vaginalis* |
| *Myosotis scorpioides* |
| *Myosoton aquaticum* |
| *Myriophyllum aquaticum* |
| *Myriophyllum spicatum* |
| *Najas minor* |
| *Nasturtium officinale* |
| *Nymphoides cristata* |
| *Nymphoides indica* |
| *Nymphoides peltata* |
| *Ottelia alismoides* |
| *Panicum repens* |
| *Phalaris arundinacea* |
| *Phragmites australis* |
| *Phyllanthus fluitans* |
| *Potamogeton crispus* |
| *Rorippa amphibia* |
| *Rotala rotundifolia* |
| *Sagittaria sagittifolia* |
| *Salvinia auriculata* |
| *Salvinia minima* |
| *Salvinia molesta* |
| *Tamarix africana* |
| *Tamarix aphylla* |
| *Tamarix canariensis* |
| *Tamarix chinensis* |
| *Tamarix gallica* |
| *Tamarix parviflora* |
| *Tamarix ramosissima* |
| *Trapa natans* |
| *Typha angustifolia* |
| *Urochloa mutica* |

| **Animals** |
| --- |
| *Acrochordus javanicus* |
| *Ameca splendens* |
| *Amphilophus citrinellus* |
| *Apocyclops dengizicus* |
| *Archocentrus nigrofasciatus* |
| *Argulus japonicus* |
| *Asellus hilgendorfii* |
| *Astatotilapia calliptera* |
| *Astronotus ocellatus* |
| *Barbonymus schwanenfeldii* |
| *Belonesox belizanus* |
| *Biomphalaria glabrata* |
| *Bithynia tentaculata* |
| *Blackfordia virginica* |
| *Bombina orientalis* |
| *Bosmina coregoni* |
| *Brachionus forficula* |
| *Bryocyclops muscicola* |
| *Bufo bufo* |
| *Bythotrephes longimanus* |
| *Caiman crocodilus* |
| *Carassius auratus* |
| *Cercopagis pengoi* |
| *Channa argus* |
| *Channa maculata* |
| *Channa marulius* |
| *Chitala ornata* |
| *Cichla ocellaris* |
| *Cichla temensis* |
| *Cichlasoma bimaculatum* |
| *Cichlasoma salvini* |
| *Cichlasoma urophthalmus* |
| *Cipangopaludina chinensis* |
| *Cipangopaludina japonica* |
| *Clarias batrachus* |
| *Colossoma macropomum* |
| *Corbicula fluminea* |
| *Cordylophora caspia* |
| *Craspedacusta sowerbyi* |
| *Ctenopharyngodon idella* |
| *Cuora flavomarginata* |
| *Cynops orientalis* |
| *Cynops pyrrhogaster* |
| *Cyprinus carpio* |
| *Danio rerio* |
| *Daphnia galeata galeata* |
| *Daphnia lumholtzi* |
| *Dendrobates leucomelas* |
| *Diaphanosoma brevireme* |
| *Diaphanosoma fluviatile* |
| *Dreissena polymorpha* |
| *Dreissena rostriformis bugensis* |
| *Drepanotrema kermatoides* |
| *Echinogammarus ischnus* |
| *Eleutherodactylus coqui* |
| *Eleutherodactylus planirostris* |
| *Eleutherodactylus portoricensis* |
| *Esox reichertii* |
| *Eunectes murinus* |
| *Eunectes notaeus* |
| *Exopalaemon modestus* |
| *Farlowella vittata* |
| *Geothelphusa dehaani* |
| *Glyptoperichthys gibbiceps* |
| *Gymnocephalus cernua* |
| *Harpacticella paradoxa* |
| *Hemichromis letourneuxi* |
| *Hemimysis anomala* |
| *Herichthys carpintis* |
| *Heros severus* |
| *Hoplosternum littorale* |
| *Hydrochoerus hydrochaeris* |
| *Hypomesus nipponensis* |
| *Hypophthalmichthys molitrix* |
| *Hypophthalmichthys nobilis* |
| *Hypostomus plecostomus* |
| *Kaloula pulchra* |
| *Lates angustifrons* |
| *Lates mariae* |
| *Lates niloticus* |
| *Leiarius marmoratus* |
| *Leporinus fasciatus* |
| *Lernaea cyprinacea* |
| *Leuciscus idus* |
| *Limnoithona sinensis* |
| *Limnoithona tetraspina* |
| *Litoria caerulea* |
| *Lophopodella carteri* |
| *Macrobrachium heterochirus* |
| *Macrognathus siamensis* |
| *Malayemys subtrijuga* |
| *Marisa cornuarietis* |
| *Mauremys caspica* |
| *Mauremys reevesii* |
| *Megacyclops viridis* |
| *Melanochromis auratus* |
| *Melanochromis johannii* |
| *Melanoides tuberculata* |
| *Melanoides turriculus* |
| *Mesocyclops ogunnus* |
| *Mesocyclops pehpeiensis* |
| *Metynnis sp.* |
| *Misgurnus anguillicaudatus* |
| *Misgurnus mizolepis* |
| *Monopterus albus* |
| *Mylopharyngodon piceus* |
| *Myocastor coypus* |
| *Myriophyllum spicatum* |
| *Natrix tessellata* |
| *Neoergasilus japonicus* |
| *Neogobius melanostomus* |
| *Nitokra hibernica* |
| *Nitokra incerta* |
| *Oreochromis aureus* |
| *Oreochromis mossambicus* |
| *Oreochromis niloticus* |
| *Oreochromis urolepis* |
| *Oreochromis urolepis hornorum* |
| *Osphronemus goramy* |
| *Osteopilus septentrionalis* |
| *Palea steindachneri* |
| *Parachromis managuensis* |
| *Paracorophium lucasi* |
| *Paracyclops bromeliacola* |
| *Paramisgurnus dabryanus* |
| *Paraneetroplus synspilus* |
| *Pelodiscus sinensis* |
| *Pelusios subniger* |
| *Petenia splendida* |
| *Pethia conchonius* |
| *Phractocephalus hemioliopterus* |
| *Physella acuta* |
| *Pisidium amnicum* |
| *Pisidium henslowanum* |
| *Pisidium moitessierianum* |
| *Pisidium supinum* |
| *Platemys platycephala* |
| *Platychirograpsus spectabilis* |
| *Platydoras costatus* |
| *Plecoglossus altivelis* |
| *Poecilia mexicana* |
| *Poecilia reticulata* |
| *Poecilia sphenops* |
| *Poeciliopsis gracilis* |
| *Pomacea bridgesi* |
| *Pomacea canaliculata* |
| *Pomacea diffusa* |
| *Pomacea haustrum* |
| *Pomacea maculata* |
| *Potamopyrgus antipodarum* |
| *Potimirim potimirim* |
| *Proterorhinus semilunaris* |
| *Pseudodiaptomus forbesi* |
| *Pseudotropheus zebra* |
| *Pterygoplichthys anisitsi* |
| *Pterygoplichthys disjunctivus* |
| *Pterygoplichthys multiradiatus* |
| *Pterygoplichthys pardalis* |
| *Pygocentrus nattereri* |
| *Radix auricularia* |
| *Rhinoclemmys pulcherrima* |
| *Rhinoclemmys punctularia* |
| *Rhinogobius brunneus* |
| *Rhodeus sericeus* |
| *Ripistes parasita* |
| *Rivulus hartii* |
| *Rocio octofasciata* |
| *Salmo letnica* |
| *Salmo trutta* |
| *Salvelinus alpinus* |
| *Sander lucioperca* |
| *Sarotherodon melanotheron* |
| *Scardinius erythrophthalmus* |
| *Sinanodonta woodiana* |
| *Sinocalanus doerri* |
| *Sphaerium corneum* |
| *Staurotypus salvinii* |
| *Stenophysa marmorata* |
| *Stenophysa maugeriae* |
| *Stephanella hina* |
| *Synodontis sp.* |
| *Systomus tetrazona* |
| *Tanichthys albonubes* |
| *Tarebia granifera* |
| *Thermocyclops crassus* |
| *Thiara scabra* |
| *Thorichthys meeki* |
| *Tilapia buttikoferi* |
| *Tilapia mariae* |
| *Tilapia rendalli* |
| *Tilapia zillii* |
| *Tinca tinca* |
| *Trichogaster trichopterus* |
| *Trichopsis vittata* |
| *Tridentiger bifasciatus* |
| *Valvata piscinalis* |
| *Viviparus viviparus* |
| *Xenopus laevis* |
| *Xiphophorus hellerii* |
| *Xiphophorus maculatus* |
| *Xiphophorus variatus* |
| *Xiphophorus xiphidium* |
