# Supplementary figures and images for: Recreational freshwater fishing drives non-native aquatic species richness patterns at a continental scale
Source: Divers Distrib. Author manuscript; Available in PMC 2018 Aug 22. (PMC6104646; doi:10.1111/ddi.12557)

FIGURE S1: SCATTERPLOT OF FRESHWATER FISHING DEMAND AND HUMAN POPULATION DENSITY.


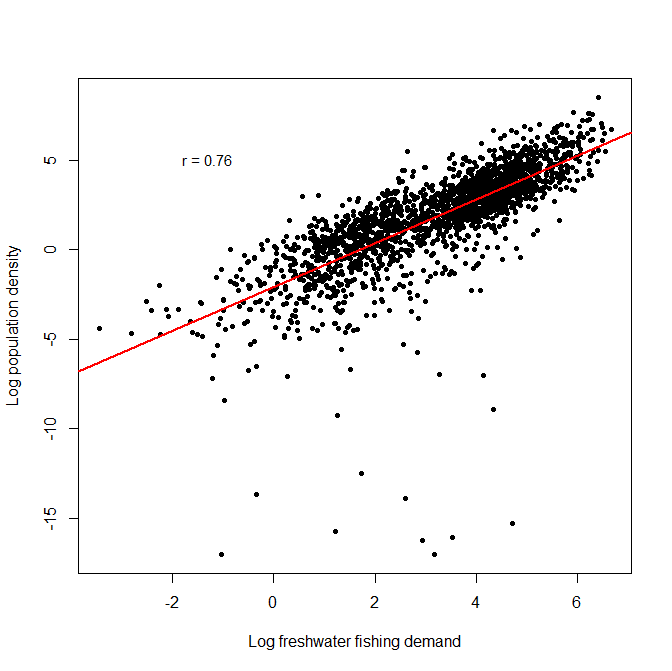

Supplement: Sup Fig1 [file NIHMS983329-supplement-Sup_Fig1.docx]
